# Preventing and Addressing the Stress Reactions of Health Care Workers Caring for Patients With COVID-19: Development of a Digital Platform (Be + Against COVID)

**DOI:** 10.2196/21692

**Published:** 2020-10-05

**Authors:** José Joaquín Mira, María Asunción Vicente, Adriana Lopez-Pineda, Irene Carrillo, Mercedes Guilabert, César Fernández, Virtudes Pérez-Jover, Jimmy Martin Delgado, Pastora Pérez-Pérez, Angel Cobos Vargas, María Pilar Astier-Peña, Olga Beatriz Martínez-García, Bárbara Marco-Gómez, Cristina Abad Bouzán

**Affiliations:** 1 Health Psychology Department Miguel Hernández University Elche Spain; 2 Alicante-Sant Joan Health District Alicante Spain; 3 The Foundation for the Promotion of Health and Biomedical Research of Valencia Region Alicante Spain; 4 Prometeo/2017/173 Excellence Group Generalitat Valenciana Valencia Spain; 5 Telematics Engineering Area Miguel Hernández University Elche Spain; 6 Andalusian Agency for Health Care Quality Seville Spain; 7 Hospital Universitario Clínico San Cecilio Granada Spain; 8 Centro de Salud La Jota Zaragoza Spain; 9 Zona de salud de Calatayud Zaragoza Spain

**Keywords:** COVID-19, pandemic, internet, social media, mobile app, psychosocial, support system, health personnel, app

## Abstract

**Background:**

COVID-19 became a major public health concern in March 2020. Due to the high rate of hospitalizations for COVID-19 in a short time, health care workers and other involved staff are subjected to a large workload and high emotional distress.

**Objective:**

The objective of this study is to develop a digital tool to provide support resources that might prevent and consider acute stress reactions in health care workers and other support staff due to the COVID-19 pandemic.

**Methods:**

The contents of the digital platform were created through an evidence-based review and consensus conference. The website was built using the Google Blogger tool. The Android version of the app was developed in the Java and XML languages using Android Studio version 3.6, and the iOS version was developed in the Swift language using Xcode version 11.5. The app was evaluated externally by the Andalusian Agency for Healthcare Quality.

**Results:**

We detected the needs and pressing situations of frontline health care workers, and then, we proposed a serial of recommendations and support resources to address them. These resources were redesigned using the feedback received. A website in three different languages (Spanish, English, and Portuguese) and a mobile app were developed with these contents, and the AppSaludable Quality Seal was granted to the app. A specific self-report scale to measure acute stress and additional tools were included to support the health care workforce. This instrument has been used in several Latin American countries and has been adapted considering cultural differences. The resources section of the website was the most visited with 18,516 out of 68,913 (26.9%) visits, and the “Self-Report Acute Stress Scale” was the most visited resource with 6468 out of 18,516 (34.9%) visits.

**Conclusions:**

The *Be + against COVID* platform (website and app) was developed and launched to offer a pool of recommendations and support resources, which were specifically designed to protect the psychological well-being and the work morale of health care workers. This is an original initiative different from the usual psychological assistance hotlines.

## Introduction

The World Health Organization (WHO) officially declared the COVID-19 outbreak a pandemic on March 11, 2020 [1]. This infectious disease was first identified in China in December 2019 and is caused by the novel coronavirus, designated SARS-CoV-2. As of June 1, 2020, a total of 5,891,182 confirmed cases had been reported worldwide. In April 2020, Spain became the second country in the world with the most reported COVID-19 cases, and as of June 1, 2020, it is the fifth country with 239,429 confirmed cases [2]. Moreover, Spain is the country with the highest rate of infections among health care workers (HCWs), representing 20.4% of all recorded cases [3].

Due to the fast spread of COVID-19 infections, the number of confirmed cases increased rapidly worldwide, and currently, it is a major public health concern [4]. As many patients with COVID-19 require hospitalization, many hospitals are saturated, and therefore, a lot of HCWs and other involved staff are being subjected to extraordinary workloads and high emotional stress [5]. Previous evidence has shown that adverse psychological reactions such as anxiety, stress, and fear were observed among health care professionals during and after other infectious disease outbreaks [6,7]. Currently, scientific studies are reporting mental health symptoms among HCWs treating patients with COVID-19 [8-10]. Cai et al [8] found that HCWs at the frontline of the COVID-19 epidemic in China were worried about the risks of infection and protective measures, resulting in psychological distress. A cross-sectional study in China showed that a significant proportion of frontline HCWs experienced anxiety, depression, insomnia symptoms, and psychological distress during the current COVID-19 pandemic [9].

To protect the psychological well-being and to prevent moral injuries of HCWs, it is necessary to better control infectious diseases [11], ensure correct functioning of the health system, and ensure that patients are receiving appropriate medical attention. Xiang et al [12] appealed for mental health care for the 2019 novel coronavirus outbreak. Health authorities must plan actions immediately to limit the impact that caring for patients with COVID-19 has on HCWs [9,10]. For the time being, evidence on how to deal with psychological distress and other mental health symptoms of HCWs who are involved in the current health crisis are not available [7], and the extent of intervention programs to assist second victims in health systems is limited [9]. However, special interventions to help frontline HCWs have been implemented in countries like China. Thus, the objective of this study is to develop a digital platform to provide support resources that might prevent acute stress reactions in HCWs and other support staff due to the COVID-19 outbreak.

## Methods

The initial step to create the platform was the development of the content and the support resources. Information about the most common concerns for HCWs during the COVID-19 outbreak and how to address them were collected through an evidence-based review and consensus conference from March 14-28, 2020. First, we carried out an evidence-based review about previous pandemics, the psychological impact of the COVID-19 outbreak on HCWs in China, and mental health care measures. Second, an online consensus conference was conducted by the principal investigator with physicians from different specialties, nurses, clinical psychologists, and responsible persons for quality and patient safety.

All members of the consensus group had experience implementing programs to support second victims.

Through the exchange of previous experiences and existing intervention results, we first identified the groups of HCWs most vulnerable to professional overload during the COVID-19 outbreak. Second, we classified the most common problem situations for HCWs. Third, we proposed possible actions to address these concerns and prevent stress symptoms.

A series of support resources were specifically designed according to the detected needs and proposed actions.

Once the platform content was defined, a website and a mobile app were developed. Due to its simplicity and efficiency in SEO positioning, the Google Blogger [13] tool was used to build the website. The blogger theme used was responsive to facilitate the usability of the web from mobile devices. The website was published in three different language versions (Spanish, English, and Brazilian Portuguese) to reach nations with common cultural characteristics. Websites were included in subdomains of the main web address of the Miguel Hernandez University, and they use a university server to store the documents available.

Concerning the app, the two most used mobile platforms were addressed: Android and iOS. According to recent reports [14], these platforms account for 99.4% of world mobile operating system market share (Android 74.14%, iOS 25.26%), so almost all mobile phone users would be able to download the app. Regarding software architecture, it was decided to develop a native app for each platform instead of creating a hybrid app valid for Android and iOS due to the better performance of native apps. As a consequence, even though the aspect and usability of both apps (Android and iOS) was the same, their internal design and the libraries used were specific for each app. The Android version of the app was developed in Java and XML languages using Android Studio version 3.6 (Google). The iOS version was developed in the Swift language using Xcode version 11.5 (Apple Inc). Both software development tools were installed on a 2017 iMac computer running macOS 10.15.4. To assure that the app met a set of quality criteria to be used in the health context, it was evaluated externally by the Andalusian Agency for Healthcare Quality through their certification program “Safety and Quality Strategy in Mobile Health Apps” [15]. This agency grants the AppSaludable Quality Seal if the app meets 31 requirements. These requirements are structured in four blocks: design and appropriateness, quality and safety of information, provision of services, and confidentiality and privacy. Fulfilling the requirements of an external and independent agency assures that the app quality and safety standards are adequate and that it can be recommended for health management. With regards to flexibility and scalability, both apps were designed with an easy to maintain structure so that future changes and additional functionalities could be carried out simply. In both apps (Android and iOS), the architecture allowed two ways of updating: (1) uploading new versions to Google Play and App Store markets and (2) refreshing the documents shown in the app or adding new documents, which could be done instantly without the need for uploading new versions and, thus, is faster and easier for the users.

Web traffic and visitor’s preferences of the website’s Spanish version as well as the app discharges were analyzed 2 months after the launch. The English and Portuguese versions of the website were launched later than the Spanish version; therefore, we considered that a longer time was needed to analyze the traffic data of these versions. Google Analytics software was used to collect the following indicators: number of visitors, number of visits, map of visitors by country, type of devices used for access, and page views. Google Analytics does not provide any personally identifiable data.

## Results

### Definition of the Digital Platform Content

Initially, the discussion group identified the most vulnerable group of HCWs to professional overload: HCWs at the frontline of COVID-19 who are in direct contact with patients with possible or confirmed infections and support staff of health care departments or units, such as the emergency department, primary health care, home hospitalization, critical care, reanimation unit, internal medicine, pneumology, and infectious diseases departments.

Taking into consideration the literature review on the SARS-CoV-2 pandemic and the opinions of the discussion group participants and their own experiences, the most common pressing situations for HCWs were identified and classified into five categories (see Textbox 1).

**Description of pressing situations for health care workers identified by category.**

**Organization, human resources, and materials**

- To be temporarily working in health care settings for which no appropriate training has been provided
  New recruitment specific for the crisis or transfers to more complex health care facilities and higher biological risk subjected to health care needs
- To receive instructions, sometimes contradictory, on the control of risks and procedures (with no clear assignment of tasks)
  Inconsistencies in the chain of command and individual proposals regarding the use of personal protective equipment, such as the use of face masks
  Shortage of material for offering the appropriate care to all patients and reducing the risk of infection
- Reduction in the number of human resources due to professionals leaving from risk exposures
  The working time is extended, the frequency of shifts increased, and the periods of physical and mental rest reduced.
  Tasks for which specific training has not been received are assumed or are carried out after express training. This may cause insecurity.
- Dissolution of stable work teams
  Incorporation of new professionals, which changes the dynamics of the work group
  Overloading of more experienced professionals
- Patients with other pathologies cannot receive the care they had been receiving.
  Over-the-phone care is provided, which increases the risk of adverse events in many cases due to the omission of actions and low probability of detecting it.
- A different perspective from residents
  They are not under the wing of the consultants but placed in a different situation regarding clinical attitude.
- Precrisis conflicts between team members
  Previous conflicts may surface now because of the task distribution in extreme situations.

**Environmental stressors and other stressors linked to crises situations**

- To work in an environment of particular biological risk
  This risk can also affect other patients, colleagues, and family members.
- To express substitutions for the loss of colleagues in isolation at home or resulting in cases with COVID-19
- To be overflown for periods that are increasing
  Wanting to not appear weak or incapable to provide an answer all the time
- No clear horizon of “how long this is going to last”

**Human factors**

- Helplessness when witnessing reckless behaviors from patients and people who accompany them (usually due to unawareness) and mistakes among professionals (from tiredness, stress, other)
  Involuntary errors are possible (which may lead to adverse events) and can be made by other professionals during patient care.
  To feel helpless, irritable, and doubting your own ability, which leads to other risk situations for the patients
- Powerlessness and disaffection at seeing how patients who are afraid of being sick with COVID-19 have to be alone; unaccompanied; and, in some cases, die in solitude. This situation is having a profound impact on professionals.

**Fear or panic reactions**

- Fear, and occasionally panic, when finding out a colleague is under passive surveillance or kept in isolation at home
- Fear of infecting a family member or a close acquaintance
- To minimize symptoms that may indicate contagion by pressure to not leave services uncovered.

**Critical decision making on health care issues**

- To be obliged to make patient triage and other decisions reserved for major catastrophes that imply relevant ethical matters
- To face the decision to prioritize levels of attention, generating a new organizational situation unknown until now

Regarding the design of interventions to prevent and handle the identified problems or pressing situations, the literature review on the experiences in Wuhan and Hunan (China) hospitals was considered. Besides, we reviewed other methods for the recovery of the second victim, which were applied in different conditions but can also be applied in this context. Finally, considering the aforementioned situations (Textbox 1), the discussion group proposed possible strategies and actions to approach the most severe demands and needs. Multimedia Appendix 1 shows the detected needs and resources to face each problem situation of HCWs during the COVID-19 outbreak. For each need, many recommendations were offered and 19 support resources were created as intervention proposals. Infographics were designed to complement some of these resources (see Multimedia Appendix 2) [16]. It is noteworthy that the nineth resource, named “self-report,” differs from the rest, and it consists of a COVID-19 acute stress scale for the HCWs. This is an ad hoc 10-question test to assess whether the HCWs were overwhelmed by the situation they were experiencing [17].

### Digital Platform Design and Development

A digital platform was developed including the aforementioned content and support resources. The name of this platform is *Be + against COVID*, and it is composed of a website in three languages (Spanish, English, and Brazilian Portuguese) and a mobile app (for Android and iOS). The site is freely accessible to anyone with an internet connection, and the app is freely available to download from the official stores.

#### Website Description

**Figure 1:**
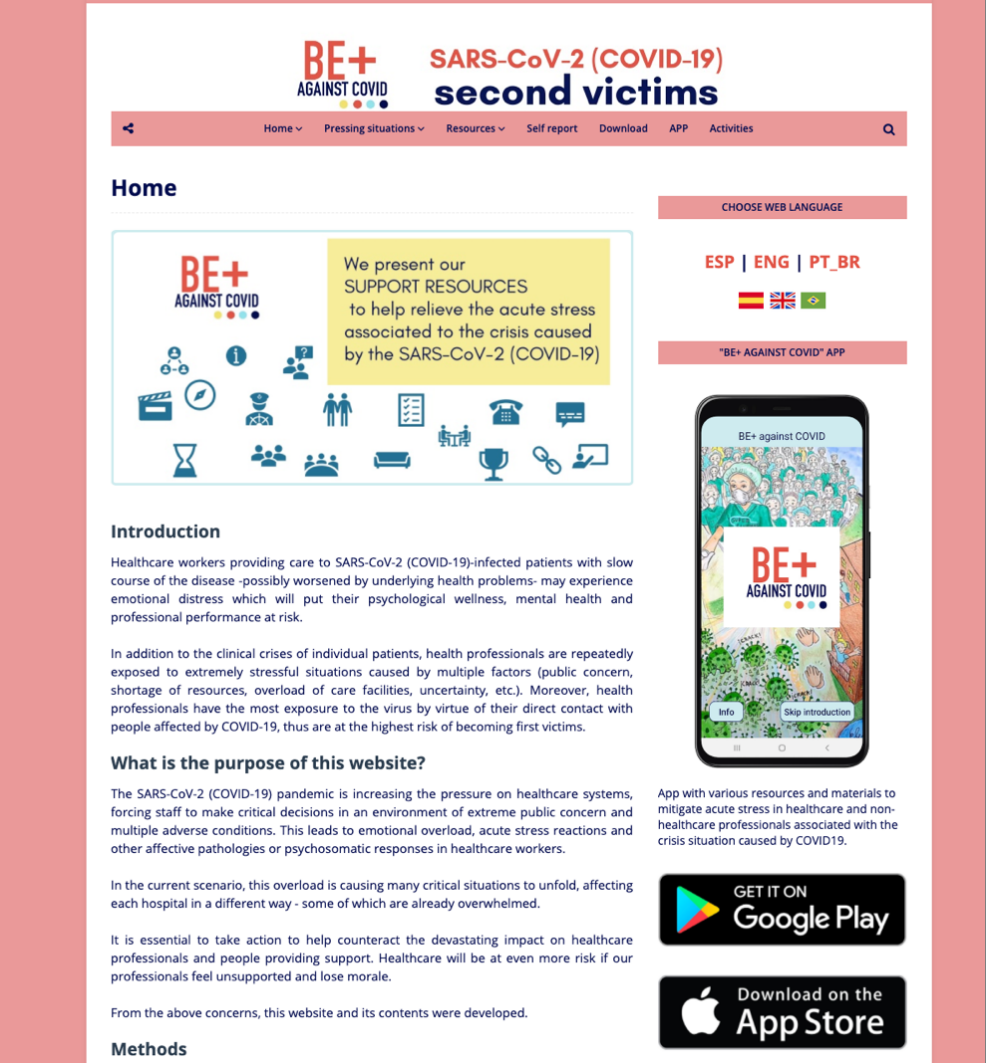
Home page and main menu from Be + against COVID website (computer view). ENG: English; ESP: Spanish; PT_BR: Brazilian Portuguese.

The three language versions share a similar content structure, although the Spanish version is the one that contains more information due to the periodic publication of news in its blog section (so-called “Positive News” [18]). Information on the website was organized following a basic structure, that is the main menu with seven items: (1) Home, (2) Pressing situations, (3) Resources, (4) Self-Report, (5) Download, (6) App, and (7) Activities. Figure 1 shows the home page of the *Be + against COVID* website. The content of each item is described in Textbox 2. The website contents were presented as text, documents (PDF format), infographics (PDF format), and videos (image format).

**Be + against COVID website content.**

- Home
  Introduction
  Purpose of this website
  Methods
  Project Team
  References
- Pressing situations
  Human factors
  Environmental stressors
  Fear or panic reactions
  Organization human resources and material
  Decision making on health care issues
- Resources
  App Be + against COVID
  Resources in short
  Resource (R)1. Information on achievements and actions taken
  R2. Involve professionals in audio-visual messages to broadcast information on guidelines (eg, safe removal of personal protective equipment)
  R3. Daily news on the situation of the center
  R4. Homogeneous structure of corporate messages
  R5. A liaison for the coordination with contract employees
  R6. Identify and refute unfounded rumors and incorrect information
  R7. Briefings at the beginning of every shift with a particular focus on new hires
  R8. Home isolation professionals to act as distant trainers and tutors for new hires
  R9. Self-report measure of acute stress
  R10. Awareness on the need to face affective response and accept support
  R11. Mental health hotline for health professionals and support by specialized personnel
  R12. Momentary work recovery (short breaks)
  R13. Defusing (face-to-face or remotely): Get rid of all emotional overload before the end of the workday to avoid taking it home and recover strength for the next shift
  R14. Referral to individual counseling to help overcome acute stress reactions
  R15. Set up rest areas for the recovery of the professionals before the end of the shift
  R16. Maintain contact and inform about the situation in the center. Long-distance institutional accompaniment. Facilitate reincorporation
  R17. Become aware of the actions expected to be carried out by middle managers: Responsible leadership
  R18. Promote informative leadership, transparency, realism, and positive messages
  R19. Make a plan to deal with the volume of delayed health care activities: Alleviate the foreseen impact on health and support professionals of this physical and mental overload
- Self Report
- Download
  Documents
  Videos focused on how to cope with distress
- App
  App information
  Download from Google Play
  Download from Appstore
- Activities

**Figure 2:**
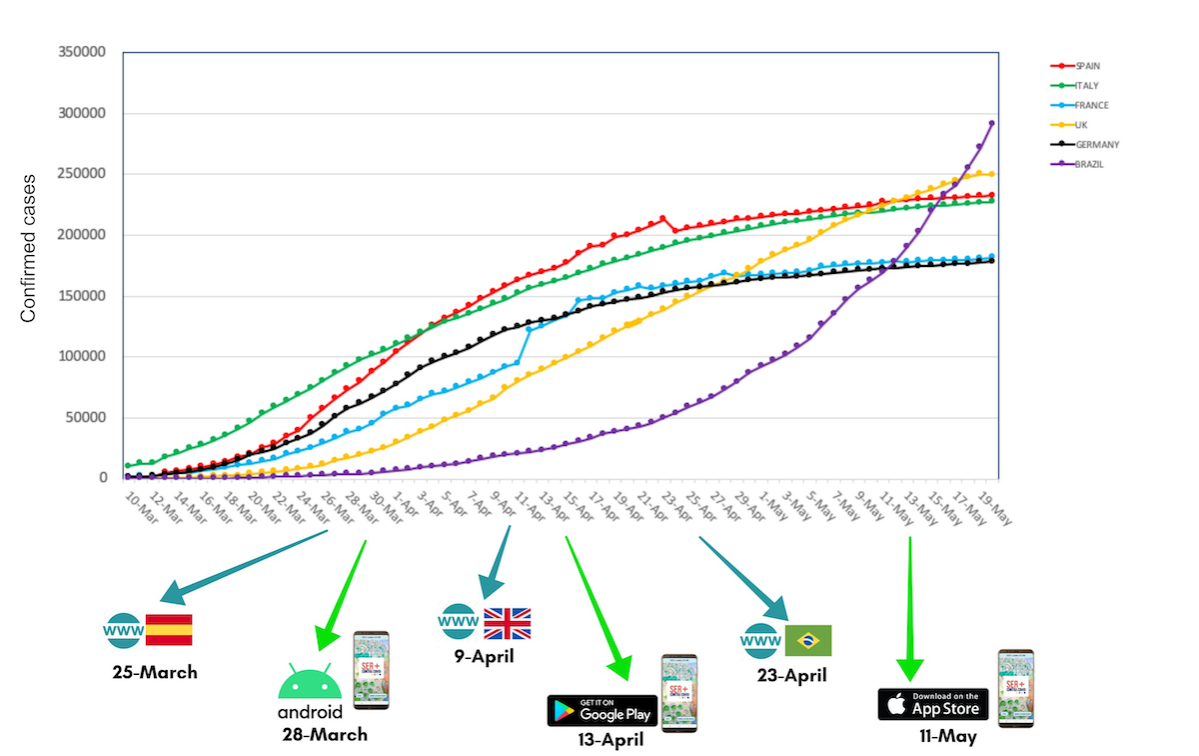
Be + against COVID platform development during the most critical phase of the SARS-CoV-2 pandemic.

The Spanish language version of the website [18] was the first to be published on March 16, 2020, because of the origin of the research team, mostly of Spanish nationality. However, it was not until March 25, 2020, that all the resources were available. The English version [19] was available from April 9, 2020, and the Brazilian Portuguese version [20] was published on April 23, 2020. Figure 2 shows the publication of each part of the platform chronologically during the most critical phase of the SARS-CoV-2 pandemic in Europe.

#### Mobile App Description

To comply with the highest quality standards, the app development process included several tests, reviews, and evaluations that modified the initial design. The first app version was developed from the requirements specified by the project team (members of the consensus group). The app was then tested internally by project members. Due to the size of the project team (with more than 20 hospitals or research centers involved), multiple comments and suggestions were received, some of them conflicting. Table 1 summarizes the main conflicting comments for the app design and the final decisions that were made. This process step resulted in a second version of the app. This version was evaluated by the Andalusian Agency for Healthcare Quality. The accreditation agency proposed several improvements that required an app redesign.

**Figure 3:**
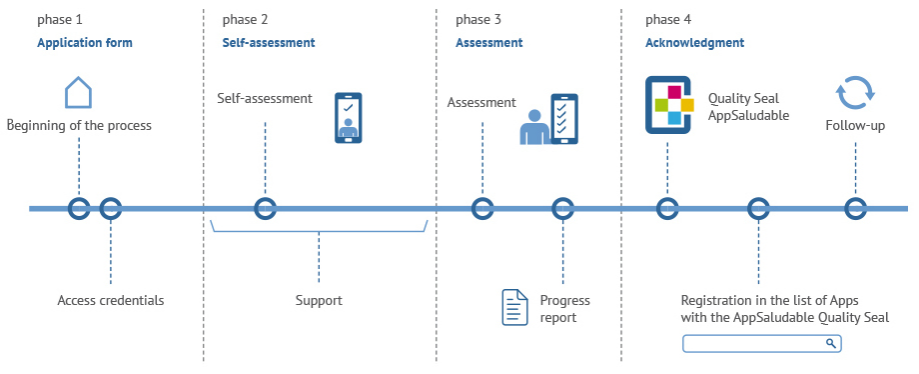
The evaluation process to obtain the AppSaludable Quality Seal.

This new version of the app was finally granted the AppSaludable (HealthyApp) Quality Seal on April 21, 2020 [21] (see Figure 3 [15]).

**Table 1:** Main conflicting suggestions during app development process.

Topic	Option 1	Option 2	Decision	Reason
Hybrid app vs native app	Hybrid to minimize developing effort	Native to improve performance in each platform (Android/iOS)	Native	Voted decision among developers
User registration	Yes to ensure reaching the correct target audience and gather statistical data	No to reinforce users’ confidence in privacy	No	Voted decision
Interface design	Colorful and optimistic to show positiveness	Neutral to show respect during a difficult situation for health professionals	Colorful and optimistic	Voted decision
External, nonproprietary documents and videos	Include them (citing sources) to offer as much information and resources as possible	Include only proprietary material to improve uniformity of style and content.	Only proprietary material included	Voted decision

**Figure 4:**
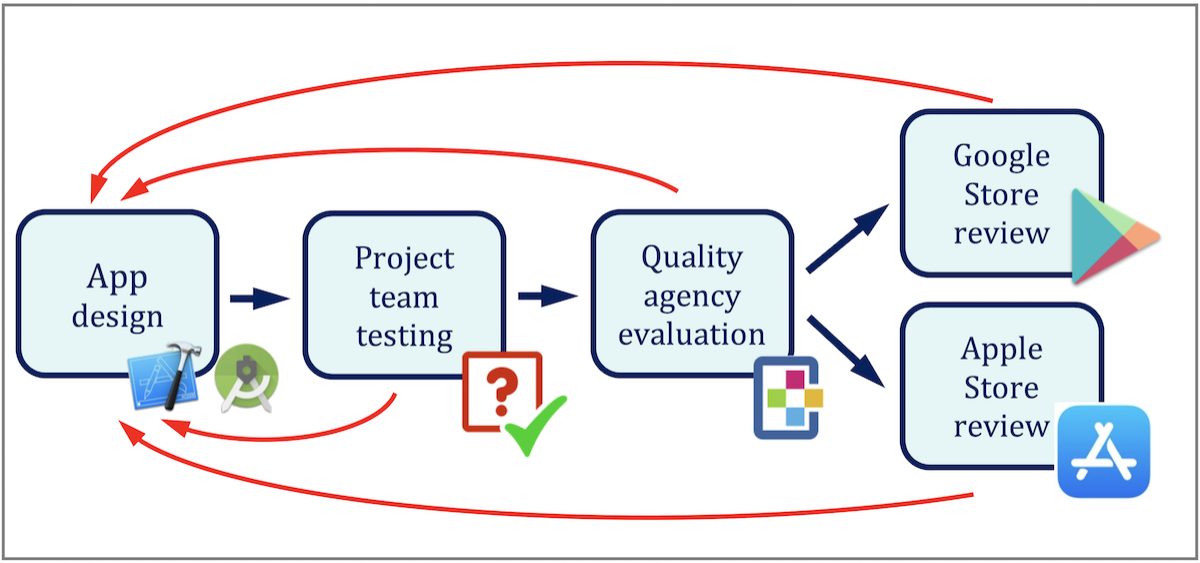
App design, test, evaluation, and review process.

Before being published in the Google and Apple stores, the app was reviewed by both companies. During the COVID-19 pandemic, both Apple and Google increased the depth of their reviews of all apps related to COVID-19. The result was a long review process with both companies, which also required small app redesigns. All redesigns were made for both platforms to keep Android and iOS versions as similar as possible. The app first appeared in Google’s Play Store on April 13, 2020, and in Apple’s App Store on May 11, 2020. As of May 31, 2020, the latest Android version is 1.8 and the latest iOS version is 1.1. Although version numbers are different, functionalities, contents, and aesthetics are the same in both apps. Figure 4 shows the complete development process of the mobile app.

The app consisted of a short presentation, which introduces the app’s purpose and provides access to an app information section. Once the introduction is complete, the app is structured into three main modules. The first module, “Advices and recommendations,” shows a list of resources that can be useful for HCWs caring for patients infected with COVID-19. These resources include posters, documents, and videos. The app was designed so that the resources were updated whenever a new version was available on the server, but at the same time, they could be accessed offline to account for possible situations where access to the internet may be limited. The second module, “Self-assessment on the ability to cope the COVID-19 crisis,” presented a 10-question test that tried to assess whether the HCWs were overwhelmed by the situation they were experiencing (resource 9 of the website). Based on the test results, the app proposed a series of guidelines or recommendations, which included in-app advice and web resources.

**Figure 5:**
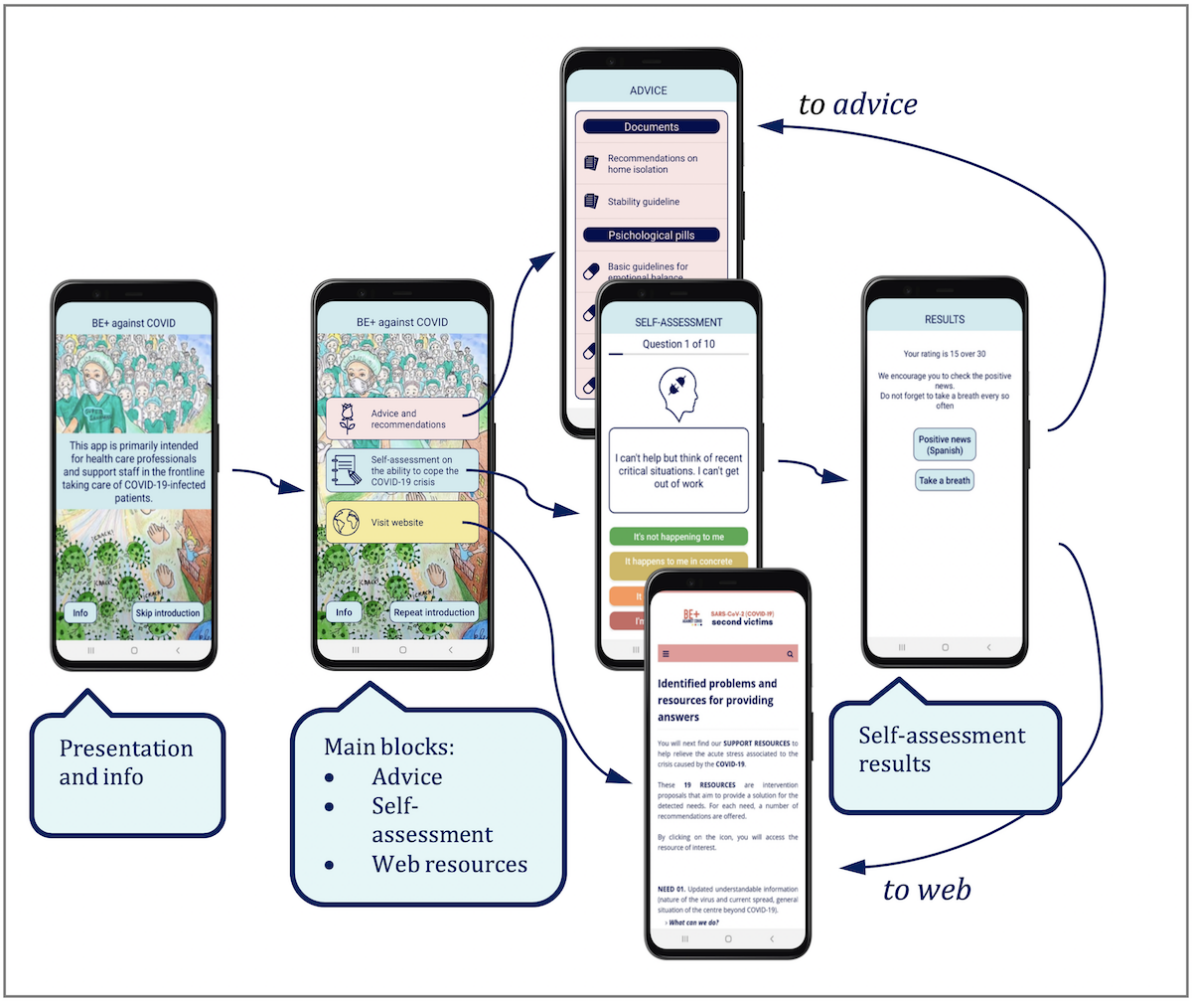
App structure explained by screenshots.

This tool underwent a process of cross-cultural and linguistic adaptation and is being currently used in studies in Argentina, Brazil, Colombia, Chile, Ecuador, and Spain, assessing the acute stress of the health care workforce during the outbreak. The third module, “Visit website,” redirected the users to the project website, where all the resources and information of the project were available. Figure 5 shows the app structure.

### Diffusion Through Social Networks

The *Be + against COVID* platform was made known to the HCWs directly through the members of the research group (see Multimedia Appendix 3), their organizations, and through the main social networks of the members and organizations involved (mainly through Facebook and Twitter). The platform’s twitter account (@second_victims) was used to further disseminate the contents of the web and the various updates and publications of the app.

No general diffusion means were used to ensure that the web and app only reached our target audience. Therefore, except for residual cases, the majority of web visits and app downloads, which will be detailed in the next sections, came from health care professionals.

### Web Analytics

#### Spanish Website Stats

From March 25, 2020 (launch of Spanish version website), to May 31, 2020, more than 7500 users visited the website in almost 70,000 different sessions. Of the 68,913 sessions, the website was accessed mostly via mobile phones with 55.8% (n=38,453) of the sessions, followed by 41.9% (n=28,875) via desktop and 2.3% (n=1585) via tablet. Users from 64 different countries accessed the website but most of the visits (n=50,058, 72.6%) were made from Spain (Table 2).

**Table 2:** List of the countries that visited the Spanish version of the website most during the most critical phase of the SARS-CoV-2 pandemic (from March 25 to May 31, 2020).

Country	Users, n	Visits, n
Spain	5612	50,058
Argentina	765	7312
Chile	284	2666
United States	214	930
Colombia	141	1499
Mexico	96	1399
Brazil	91	1262
Others	493	3787

Table 3 shows the traffic distribution to the website in the same period, and the most viewed pages of the 68,913 sessions were the home page, with 43.2% (n=29,763) of the sessions, and resources, with 26.9% (n=18,516) of the sessions. The most viewed resource was the self-report questionnaire (resource 9; see Table 4).

**Table 3:** Traffic distribution to the Spanish version of the website (percentage of visits to webpages from March 25 to May 31, 2020).

Category	Web contents	Visits (N=68,913), n (%)
Home	Introduction, purpose, methods, project team, and references	29,763 (43.2)
Resources	Resources in short and the 19 detailed resources	18,516 (26.9)
Other	Download, Documents, Videos, Activities, HCWs^a^ agreements, Tell us your experience, etc	7003 (10.2)
App	App information, manual, and links to the official stores	5695 (8.3)
Problems	Pressing situations, human factors, environmental stressors, fear or panic reactions, organization, human resources, material, and decision making	4067 (5.9)
Positive news	Posts on positive news related to the SARS-CoV-2 pandemic	3869 (5.6)

^a^HCW: health care worker.

**Table 4:** Visits to resources pages (Spanish version of the website) from March 25 to May 31, 2020.

Resources’ contents	Visits (N=18,516), n (%)
All resources in short	3062 (16.5)
R^a^1 Achievements	766 (4.1)
R2 Informative videos	683 (3.7)
R3 Daily News	528 (2.9)
R4 homogenous messages	376 (2.0)
R5 Contract employees	304 (1.6)
R6 Fake News	248 (1.3)
R7 Briefings	624 (3.4)
R8 Long distance trainers	399 (2.2)
R9 Self-report	6468 (34.9)
R10 Emotional coping	1112 (6.0)
R11 Hotline support	459 (2.5)
R12 Recovery Time	736 (4.0)
R13 Defusing	785 (4.2)
R14 Referral to a professional	327 (1.8)
R15 Resting areas	413 (2.2)
R16 Contact during confinement	248 (1.3)
R17 Responsible leadership	254 (1.4)
R18 Informative leadership	241 (1.3)
R19 Post crisis	485 (2.6)

^a^R: resource.

#### App Stats

**Figure 6:**
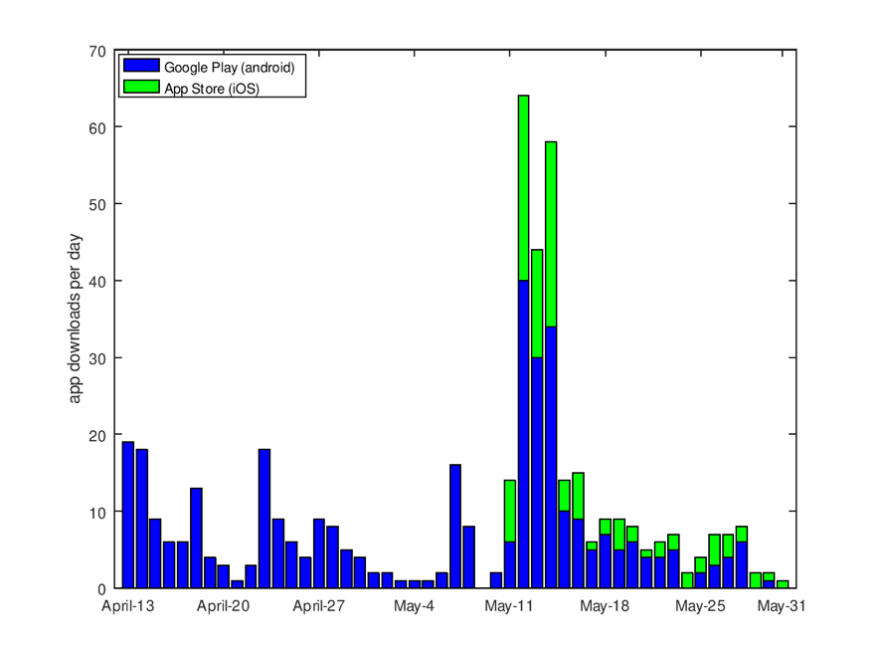
Daily app downloads from official app stores.

Figure 6 shows the number of daily app downloads from Google Play (Android version) and the App Store (iOS version). The Android version was first published on Google Play on April 1, 2020, while the iOS version was first published on May 11, 2020. There were 472 total app downloads during this period (Android: n=361; iOS: n=111). App downloads from our server, which started on March 28, 2020 (before the publication in the official app stores), were not included in these figures.

Regarding the self-assessment test, a total of 388 tests were completed from the app as of May 31, 2020. This number increased according to the intensity of the outbreak and particularly in territories where incidence and mortality of COVID-19 was higher.

A simple statistical analysis was carried out using the data gathered from app users who took the self-assessment test (n=388). After showing the test results, the app offers the users different choices depending on the results. Most users (n=204, 52.5%) opted for browsing the list of psychological pills and reading about one of these pills, followed by 21.2% (n=82) of users who decided to have a look at the positive news and 18.6% (n=72) of users who opted for reading instructions on how to take a breath. The remaining 7.7% (n=30) of users chose to access the resources for overcoming acute stress reactions.

A more in-depth analysis showed the user engagement capability of the app. Using device identifiers to detect repeated user accesses to the app self-assessment test, we found that most of the 388 users (n=246, 63.4%) carried out the test only once, but 36.6% (n=142) of users took the survey more than once on different dates. More in detail, 14.7% (n=57) of the users took the test twice, 11.1% (n=43) three times, 2.8% (n=11) four times, and 8.0% (n=31) five or more times. Concerning the time spent from the first and last self-assessment carried out by the same user (long-term engagement), 2.1% (n=8) of the users took the test on different dates separated by at least 2 months, 6.3% (n=24) at least 1 month, 13.4% (n=51) at least 15 days, and 16.2% (n=63) at least 1 week.

Given that the app was published just a few weeks ago, the number of results is still small; however, the data collected offers interesting information on user behavior.

## Discussion

### Principal Results

This paper shows the development and the start-up of a project that arose from the urgent need to prevent and address the pressing situation that HCWs were and are facing in the world. Since Spain has been one of the first and most affected countries by the COVID-19 outbreak worldwide, this project can serve as an experience for others. The *Be + against COVID* platform (website and mobile app) was developed in three language versions to guide on possible actions that can be put into practice in health care centers to help confront the emotional impact on frontline HCWs caused by the current pandemic. Since there was no background about similar initiatives, the experience of China, previously published initiatives to facilitate the recovery of the second victim, and the interventions already implemented in some hospitals in Spain were considered to plan prevention and approach actions. During the first 2 months from the launch of the Spanish language version of the website, users from different countries accessed the website, but visitors were mostly from Spain. The most interesting and useful part of the website for HCWs was the resources section composed by intervention proposals that aim to provide a solution for the detected needs, and the “self-report” was the most visited resource. Currently, the Spanish language version of the website is being updated with content related to the recovery phase (postcrisis).

These resources were designed to help address the emotional consequences of caring for patients with COVID-19 in the context of the outbreak. This situation was characterized by a lack of foresight, demand for an immediate response from health professionals, and a shortage of resources. The focus was on providing resources through this platform for individual use.

Professionals from different disciplines (psychologists, psychiatrists, occupational health professionals, clinicians, and nurses) were involved in the design and redesign of these resources to support frontline health professionals in the care of patients with COVID-19, gathering experience in many hospitals and health care centers. They have assessed the adequacy of resources to the needs experienced by the professionals and have suggested improvements. In addition, Latin American professionals have been involved in the transcultural and linguistic adaptation of the resources and the self-report scale. Given that the expansion of the outbreak has been different according to each territory, the proposal of resources to face acute stress must be wide, varied, and flexible. The data confirm that acute stress increases in those territories with the greatest number of cases and highest mortality. In this case, the acceptability of the proposal is greatest [22]. In other countries, some hospitals provide psychological assistance hotlines to HCWs and carry out prevention programs. To offer a pool of recommendations and support resources through a free website is a different and original initiative. To the best of our knowledge, there is no similar experience in Europe or Latin America. We consider that this paper may be useful to develop a web-based tool with a similar objective or in a similar context. The Brazilian research group Enfermagem da Escola Paulista de Enfermagem da Universidade Federal de São Paulo (EPE-UNIFESP) translated our resources into Portuguese, and they were incorporated into its institutional website [23].

### Limitations

Given the current situation, conditions may differ across time and context, and therefore, our proposal of interventions is limited to the time and context of this study. These proposed actions can change due to the urgency and demand of the situation. Additionally, the response of HCWs to the COVID-19 crisis might be different between countries, and therefore, the problem situations and resources may be applicable in health care systems of countries with similar sociocultural characteristics. The urgency of the situation did not allow us to analyze the effectiveness of the proposed interventions. Future research should investigate it.

### Comparison With Prior Work

Psychological assistance has been provided through telephone-, internet-, and application-based interventions to the health care community by some local or national institutions in response to the COVID-19 outbreak [9]. Several international organizations such as the WHO, the United Nations, and the International Red Cross Society have published recommendations that team leaders can consider to reduce stress and the psychological distress of frontline HCWs. Social support, clear communication, flexible working hours, and psychological help are some of these recommendations [24]. The State Council of China developed a psychological intervention plan, which consists of setting up nationwide psychological assistance hotlines to help during the outbreak [11,25]. However, some workers did not recognize any problems and refused any psychological help [11]. The Canadian Medical Association also offers some resources for supporting HCWs that focus on psychological assistance provided by volunteer psychologists [26]. On the other hand, Blake et al [27] developed and evaluated a digital learning package that includes evidence-based guidance, support, and signposting regarding psychological well-being for frontline HCWs from the United Kingdom. This online support package is free and comprised of 88 slides, and its content is similar to that of the digital platform developed by our team. Similar to our digital platform, this electronic package describes the actions that team leaders can take to support HCWs during the COVID-19 outbreak and offers some support resources (infographic and video formats) to protect psychological well-being [27]. The evaluation showed that it is useful and appropriate for any UK health care professional. In contrast to our website, which was published in three different language versions, Blake et al [27] seemed to be focused on workers from the United Kingdom to develop this package. Moreover, nowadays, a mobile app can be more practical for some users. Nevertheless, the fidelity, engagement, usability, and practicality of our digital platform should be evaluated in future research.

### Extrapolation to Other Contexts

Although there are differences between the health systems of the different countries and it is evident that the spread of the SARS-CoV-2 pandemic is different in each continent and country, most of the problems identified, and for which these resources have been devised, are repeated in the literature [9,28-30], and it is verified in the experience described by health professionals in Brazil, Peru, Argentina, Colombia, Mexico, Ecuador, Italy, Portugal, and Central Europe.

The extrapolation of resources to mitigate acute stress among frontline health professionals in the care of patients with COVID-19, therefore, could be carried out in both public and private centers.

The development and redefinition of resources to support the health care workforce and address new outbreaks must take into account differences in organizational culture [31-33], psychological safety [34], and availability of financial and skilled human resources among countries.

Although similarities have been observed in the sources and acute stress responses of these professionals, there is a need to adapt resources to the context as, for example, has been done in Brazil, adapting the resources originally designed for the Spanish National Healthcare System. Future research studies may consider these differences when assessing the applicability of the resources designed.

### Upcoming Developments

Preparing for a spike in new COVID-19 cases from health care organizations and professionals, particularly on the frontline of care for patients with COVID-19, should be prioritized by health planners and managers, academics, and health care professionals. Systems have more resources and better organization and information to reduce clinical uncertainty, but templates need to be recovered physically and emotionally. The new developments of this platform are incorporating proposals to strengthen the resilience of organizations and professionals.

### Conclusions

A digital platform (website and app) was developed and launched to provide support resources that might prevent and approach the emotional impact of HCWs and other support staff due to the current COVID-19 outbreak.

## Appendix

Multimedia Appendix 1 Identified problem situations of health care workers during the COVID-19 outbreak, generated needs, and available resources for an appropriate response.

Multimedia Appendix 2 Infographics of some of the resources from the Be + against COVID platform.

Multimedia Appendix 3 Project team.

## Abbreviations

EPE-UNIFESP: Enfermagem da Escola Paulista de Enfermagem da Universidade Federal de São Paulo
HCW: health care worker
IRYCIS: Instituto Ramón y Cajal de Investigación Sanitaria
SECA: Sociedad Española de Calidad Asistencial
WHO: World Health Organization
