# Supplementary material for: Preventing and Addressing the Stress Reactions of Health Care Workers Caring for Patients With COVID-19: Development of a Digital Platform (Be + Against COVID)
Source: JMIR Mhealth Uhealth. 2020 Oct 5;8(10):e21692. doi: 10.2196/21692 (PMC7537725; doi:10.2196/21692)
Supplement: Multimedia Appendix 1 [file mhealth_v8i10e21692_app1.doc]

Supplemental file 1. Identified problem situations of healthcare workers during the COVID-19 outbreak, generated needs and available resources for an appropriate response.

| **PROBLEM SITUATION** | **Need (NE)** | **Resource (RE)** |
| --- | --- | --- |
| **ORGANIZATION, HUMAN RESOURCES AND MATERIALS** | | |
| **Uncertainty** regarding the situation caused by the COVID-19 crisis | **NE1**. Updated and understandable information (evolution of the pandemic, general status of the center beyond COVID-19) | RE1. Information on achievements and performed actions  RE2. Get professionals involved in audio-visual messages to transmit information on guidelines (e.g., correct doffing of personal protective equipment) |
|  | **NE2**. Information on available human resources, supplies and healthcare equipment | RE3. Announcement on the situation of the center on a daily basis |
|  | **NE3**. Visualize coordination, unity of criteria, day-to-day normalization despite being in the epicenter of the crisis | RE4. Homogeneous structure of corporate messages  RE5. Coordination link for new hires  RE6. Identify and refute rumours and incorrect information |
| Be working temporarily in **healthcare setting for which no appropriate training has been received** either because of newly hired personnel or because of a transfer to more complex healthcare settings, which promotes **insecurity and acute stress**. | **NE4**. Offer safety to the newly hired professionals and to those who have been transferred to other services | RE7. Briefings at the beginning of every shift with guidance to newly arrived staff  RE8. Professionals in home confinement as distant trainers and tutors of new professionals |
|  | **NE5**. Relief and emotional support to overflowed personnel | RE9. Self-assessment of acute stress responses  RE10. Awareness on the need to face emotional response and accept support  RE11. Hotline for psychological support to healthcare and support professionals by specialized personnel  RE12. Recovery time (short breaks) during the workday |
| Change of **instructions** on organizational issues **and** procedures, added to **inconsistencies in the chain of command** caused by the rapid variations that occur in all crises | **NE2**. Information on available human resources, supplies and healthcare equipment | RE3. Announcement on the situation of the center on a daily basis |
|  | **NE3**. Visualize coordination, unity of criteria, day-to-day normalization despite being in the epicenter of the crisis | RE4. Homogeneous structure of corporate messages  RE5. Coordination link for new hires  RE6. Identify and refute rumours and incorrect information |
|  | **NE9**. Strengthen the leadership capacity | RE17. Become aware of the actions expected to be carried out by middle managers. Responsible leadership  RE18. Promote informative leadership, transparency, realism and positive messages |
| **Reduction of human resources** due to leave of professionals with risk exposures. This forces to **extend workdays**, increase the frequency of shifts and reduce physical and mental **break times**  **Express substitutions** due to home isolation or medical leave of colleagues | **NE2**. Information on available human resources, supplies and healthcare equipment | RE3. Announcement on the situation of the center on a daily basis |
|  | **NE5**. Relief and emotional support to overflowed personnel | RE12. Recovery time (short breaks) during the workday |
|  | **NE8**. Professionals confined to their homes with feelings of impotence for the situation their colleagues are going through | R16. Maintain contact and inform about the situation in the center. Long-distance institutional accompaniment. Facilitate reincorporation |
| **Dissolution of stable work teams** due to the incorporation of new professionals, overloading most experienced professionals | **NE3**. Visualize coordination, unity of criteria, day-to-day normalization despite being in the epicenter of the crisis | RE4. Homogeneous structure of corporate messages |
|  | **NE4**. Offer safety to the newly hired professionals and to those who have been transferred to other services | RE7. Briefings at the beginning of every shift with guidance to newly arrived staff  RE8. Professionals in home confinement as distant trainers and tutors of new professionals |
|  | **NE9**. Strengthen the leadership capacity | RE17. Become aware of the actions expected to be carried out by middle managers. Responsible leadership  RE18. Promote informative leadership, transparency, realism and positive messages |
| Patients with **other pathologies (non-COVID-19)** who do not receive the attention they had received before the crisis due to new healthcare priorities | **NE1**. Updated and understandable information (evolution of the pandemic, general status of the center beyond COVID-19). | RE1. Information on achievements and performed actions |
|  | **NE3**. Visualize coordination, unity of criteria, day-to-day normalization despite being in the epicenter of the crisis | RE4. Homogeneous structure of corporate messages |
| **Pre-crisis conflicts between team members** that may surface now because of the task distribution in this extreme situations | **NE4**. Offer safety to the newly hired professionals and to those who have been transferred to other services. Strengthening of new work teams. | RE7. Briefings at the beginning of every shift with guidance to newly arrived staff. Strengthen team unity. |
| **HUMAN FACTORS** | | |
| Helplessness and irritability when witnessing **reckless behaviors from patients and people who accompany them** (usually due to unawareness) and **healthcare mistakes** (from tiredness, negative emotions, etc.). **Involuntary errors** that may lead to adverse consequences.  Face the **anxiety of strictly isolated** hospitalized COVID-19 patients and watching how some die alone | **NE5**. Relief and emotional support to overflowed personnel | RE9. Self-assessment of acute stress responses  RE10. Awareness on the need to face emotional response and accept support  RE11. Hotline for psychological support to healthcare and support professionals by specialized personnel  RE12. Recovery time (short breaks) during the workday |
|  | **NE6**. Relief and emotional support to the personnel from services and units undergoing extreme stress | RE13. Defusing (face-to-face or at a distance). Get rid of all emotional overload before the end of the workday to avoid taking it home and recover strength for the next shift |
| **Environmental stressors and other stressors linked to crisis situations** | | |
| A work situation of special **biological risk**. This risk may imply infecting **patients, colleagues and family members** you live with at home | **NE7**. Recover professionals beset by doubts and fears due to the risk of being exposed or who feel emotionally overwhelmed | RE14. Referral to individual counselling to help overcome acute stress reactions  RE15. Set up rest areas for the professionals to recover before the end of the shift |
| Be **overwhelmed** by events for moments that become larger **without being able to talk about it** to not look weak | **NE5**. Relief and emotional support to overflowed personnel | RE11. Hotline for psychological support to healthcare and support professionals by specialized personnel |
|  | **NE6**. Relief and emotional support to the personnel from services and units undergoing extreme stress | RE13. Defusing (face-to-face or at a distance). Get rid of all emotional overload before the end of the workday to avoid taking it home and recover strength for the next shift |
|  | **NE7**. Recover professionals beset by doubts and fears due to the risk of being exposed or who feel emotionally overwhelmed | RE15. Set up rest areas for the professionals to recover before the end of the shift |
| **No clear horizon** of “how long this is going to last” | **NE1**. Updated and understandable information (evolution of the pandemic, general status of the center beyond COVID-19). | RE1. Information on achievements and performed actions |
|  | **NE2**. Information on available human resources, supplies and healthcare equipment | RE3. Announcement on the situation of the center on a daily basis |
|  | **NE5**. Relief and emotional support to overflowed personnel | RE9. Self-assessment of acute stress responses  RE10. Awareness on the need to face emotional response and accept support |
| **FEAR OR PANIC REACTIONS** | | |
| **Fear** when finding out that a colleague is in **passive surveillance** or **home isolation**  **Fear** of infecting family members and/or close friends | **NE7**. Recover professionals beset by doubts and fears due to the risk of being exposed or who feel emotionally overwhelmed | RE14. Referral to individual counselling to help overcome acute stress reactions |
| **CRITICAL DECISION-MAKING REGARDING HEALTHCARE ISSUES** | | |
| Be obliged to make **patient triage** and other decisions reserved for major catastrophes that imply relevant **ethical matters**. | **NE5**. Relief and emotional support to overflowed personnel | RE9. Self-assessment of acute stress responses  RE10. Awareness on the need to face emotional response and accept support  RE11. Hotline for psychological support to healthcare and support professionals by specialized personnel |
|  | **NE6**. Relief and emotional support to the personnel from services and units undergoing extreme stress | RE13. Defusing (face-to-face or at a distance). Get rid of all emotional overload before the end of the workday to avoid taking it home and recover strength for the next shift  RE15. Set up rest areas for the professionals to recover before the end of the shift |
|  | **NE9**. Strengthen the leadership capacity | RE18. Promote informative leadership, transparency, realism and positive messages |
| **POST-CRISIS** | | |
| **Post-crisis** | **N10**. Manage the post-crisis by taking the initiative and be one step ahead SARS-CoV-2 | RE19. Make a plan to deal with the volume of delayed healthcare activities. Alleviate the foreseen impact on health and support professionals of this physical and mental overload |
