# Supplementary material for: Preventing and Addressing the Stress Reactions of Health Care Workers Caring for Patients With COVID-19: Development of a Digital Platform (Be + Against COVID)
Source: JMIR Mhealth Uhealth. 2020 Oct 5;8(10):e21692. doi: 10.2196/21692 (PMC7537725; doi:10.2196/21692)
Supplement: Multimedia Appendix 2 [file mhealth_v8i10e21692_app2.pdf]

DEFUSING IN HEALTHCARE CENTRES

COVID-19 CRISIS

GET RID OF ALL EMOTIONAL OVERLOAD BEFORE THE END OF THE WORKDAY TO AVOID TAKING IT HOME AND RECOVER STRENGTH FOR THE NEXT SHIFT

WHAT IT IS?

1. A strategy to share emotions and experiences
2. After being exposed to situations of extreme stress
3. May become part of the daily routine for the different teams (15-10')

DON'T CONDUSE DEFUSING WITH:

1. Group therapy
2. Critical analysis of errors and improvement actions
3. A search of responsibilities or exchange of reproaches

WHY DO WE DO IT?

- To **deactivate** discomfort and tension
- To **recover** strength and confidence
- To **avoid** taking stress back home and be able to take a break
- To **create** an atmosphere of mutual support, enhance team unit
- To **preserve** well-being
- To **prevent** extreme stress responses and errors caused by emotional overload
- To **integrate** adaptively critical experiences of the workday
- To **detect** if I am emotionally overloaded and need some additional help.

VENT • CLOSE • RESTORE • CONTINUE

WHO?

- **Small groups** (6-10 people) - Personnel from the unit present in the shift
- **Moderator:** person responsible of patient's or the unit's safety, prevention of occupational hazards or mental health personnel, external staff

WHEN AND WHERE?

At the end of the workday

In a place where little interruption occurs

Face-to-face or streaming

WHAT TO COMMENT?

ADAPTABLE TO THE SITUATION

How was the workday?

How are you doing?

What difficult situation did you deal with today?

How are you feeling?

What worries you the most?

What repetitive thoughts have you had throughout the shift?

Have your experiences today changed the way you see things?

Which has been the worst moment during this shift?

What has impacted/affected you the most?

Is there any task/decision you used to handle easily before which you had little strength to do today?

Is there anything else you would like to comment?

Does anyone else share these feelings/worries?

OTHER INFORMATION OF INTEREST

Voluntary participation

Confidentiality of shared information

Time for all to speak

End the session with positive messages of unit and strength

BE+ AGAINST COVID

https://secondvictimscovid19.umh.es/

TIPS TO FIGHT AGAINST
COVID-19 FAKE NEWS

TO IDENTIFY AND FIGHT AGAINST FAKE NEWS THE FOLLOWING SHOULD BE CONSIDERED

That the provided information is always received from the same **official source**, such as governments, research and science institutions, or health-focused organizations.

That the health-related information be preferably in **video** format, as this type of messages are more credible in comparison to other means, such as voice or text messages. Video messages allow easier identification of the source.

That informative chain messages in messaging services (e.g., WhatsApp) are linked to **official accurate accounts**, i.e., avoiding to attach the information directly (text, voice message, video, etc.) from unknown sources.

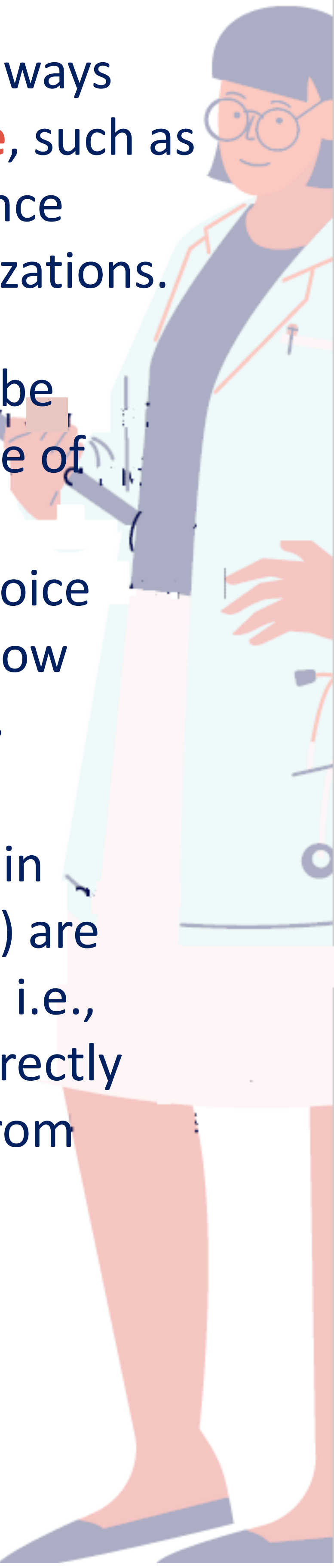

BE+
AGAINST COVID

https://secondvictimscovid19.umh.es/

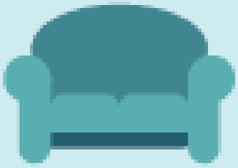

JACOBSON´S BRIEF MUSCLE RELAXATION

TECHNIQUE AGAINST ANXIETY AND STRESS

1 **Place yourself in a comfortable position: SITTING**
Leave your mind in blank
A calm environment will also help

2 **Tighten the muscles of the FOREHEAD**
To achieve this, rise your eyebrows for six seconds. Next, relax your forehead for 15 seconds and focus in the sensation of relaxation

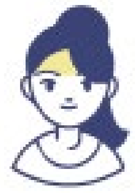

3 **Tense the EYES and NOSE AREA**
Tighten the eyelids and wrinkle the nose for six secondsand release the tension for 15 seconds. Feel what happens

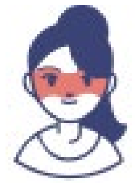

4 **Tighten the muscles of the MOUTH**
Next, you have to grit your teeeth, lips and tongue against the palate for six additional seconds and relax for 15 seconds

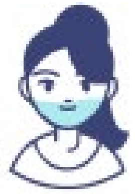

5 **Tighten your neck NECK MUSCLES**
Grit your teeth, lips and tongue against the palate for six additional seconds and relax for 15 seconds

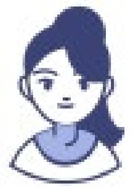

6 **REPEAT THE PROCESS AS MANY TIMES AS YOU NEED TO DO SO**
Remember you can accompany this exercise with breathing techniques

It is important to **be patient** and ifrelaxation is not achieved the first time, it´s a matter of **practice**

BE+ AGAINST COVID
SARS-CoV-2 (COVID-19) second victims
https://secondvictimscovid19.umh.es

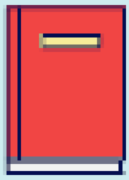

MY DIARY OF POSITIVE NOTES

ONE MINUTE EACH DAY TO BRING OUT THE POSITIVE OF THE PRESENT SITUATION

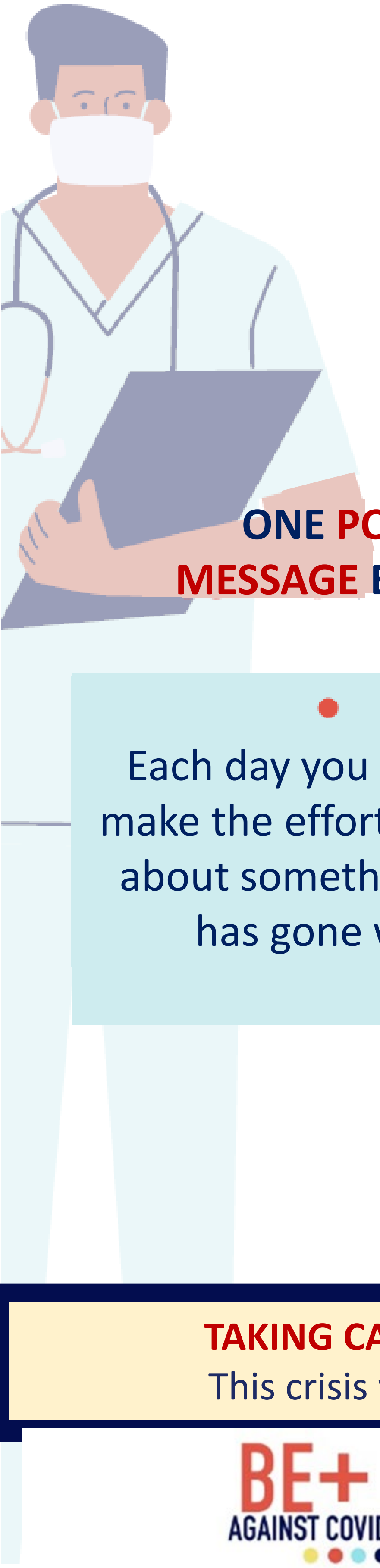

GET SOMETHING TO WRITE ON

It can be a notebook, a sheeet of paper, or post-it notes to place on the fridge or the new board in the floor you work

ONE POSITIVE MESSAGE EVERY DAY

Each day you have to make the effort to think about something that has gone well

THIS WILL HELP your mind KEEP FOCUSED ON THE POSITIVE SIDE

You will be able to balance all achievements and challenges occurring throught the day

TAKING CARE of your MIND is ALSO IMPORTANT
This crisis **will test us physically and emotionally**

BE+ AGAINST COVID

SARS-CoV-2 (COVID-19) second victims

https://secondvictimscovid19.umh.es

TAKE A BREAK AGAINST STRESS

THE two-minute STOP TECHNIQUE

S

FEEL How you keep still wherever you are

By doing this, you are deciding to take control instead of allowing external pressure to take over you and make you react and stress.

T

Take a few minutes to observe your body

Slowly scan your body beginning by the tip of your toes working your way up to the top of your head. As you move up, notice where your tensions or negative emotions acumulate. Breathe towards the tension and then let go

O

CLOSELY OBSERVE what surrounds you

Look around you for small details you have never observed before. They should be nice for you to see. Inmerse and become part of your surrounding until you find something you like or surprises you

P

POSSIBILITIES you now have

You have just turned off autopilot mode and are free to choosea new more beneficial direction. If you were feeling overloaded, exhausted, and not knowing what to do, you can now see a variety of diferente choicesand options in front of you with fresh eyes...

HOW WAS YOUR EXPERIENCE WITH THE STOP YOU HAVE JUST MADE?

Repeat the sequence **several times a day** to **relieve** emotional tension, take better care of yourself, patients and close ones.

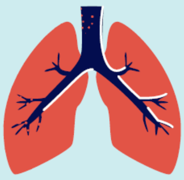

DIAPHRAGMATIC BREATHING
TAKE A BREATH

REMEMBER THE REASONS FOR MAKING A STOP

IT RELAXES YOU	IT PROTECTS YOU	IT BALANCES YOU
Allows releasing anxiety and stress	Decreases over-activation	Favours homeostatic regulation

HOW TO DO THIS?

1 **WHEN TAKING A BREATH, PLACE ONE HAND ON THE CHEST AND THE OTHER ON YOUR STOMACH**
Make sure you let the air towards the lower part of the lungs without moving your chest

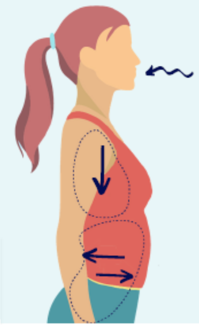

2 **AS YOU BREATH IN, ALLOW YOUR STOMACH AND BELLY TO RISE WITHOUT MOVING YOUR CHEST**

3 **RETAIN THE AIR, FOR A MOMENT WHILE IN THIS POSITION**

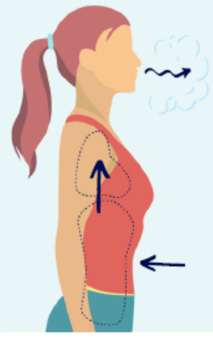

4 **RELASE THE AIR SLOWLY, TIGHTENING YOUR ABDOMEN SLIGHTLY WITOUT MOVING THE CHEST**

5 **TRY TO REMAIN RELAXED FOR FOUR SECONDS WITH YOUR LUNGS EMPTY UNTIL THE NEXT INHALATION**

BE+ AGAINST COVID

SARS-CoV-2 (COVID-19) second victims

https://secondvictimscovid19.umh.es

RAPID ON-LINE RESOURCES

FOR PROFESSIONALS IN ISOLATION WHO ARE ACTING AS TRAINERS

WHATSAPP

DOUBTS-ANSWERS FOR TUTORS OR SUPPORT PERSONNEL

- It allows up to four people to interact simultaneosly in a video call
- Information can be shared directly through the audio, video or images
- Most people have a smartphone and the WhatsApp app

GOOGLE HANGOUTS

IT ALLOWS PERFORMING DIRECT VIDEO CONFERENCING OR A QUESTIONS AND ANSWERS SESSION BETWEEN TUTORS AND SUPPORT STAFF

- The user inviting to the video conference must have a Gmail account
- It can be used in PC and smartphones
- Possible participation of a large number of people

RAPID ON-LINE RESOURCES

FOR PROFESSIONALS IN ISOLATION WHO ARE ACTING AS TRAINERS

LIFESIZE

TO ORGANIZE ONLINE MEETINGS HOSTING UP TO 25 PARTICIPANTS BETWEEN TUTORS AND SUPPORT STAFF

- The user inviting the meeting must register in the LIFESIZE platform (free trial version)
- Can be used on **any device**
- Allows real-**time screen** and document sharing
- **High-quality video**

SKYPE

GROUP VIDEO CALLS WITH UP TO 50 TUTORS AND SUPPORT STAFF

- The user inviting the meeting as well as the guests must have a **Skype account**
- Allows real-time **presentation of slides, sharing documents**, videos or recording
- Classic tool for group video calls

BE+ AGAINST COVID

SARS-CoV-2 (COVID-19) second victims

https://secondvictimscovid19.umh.es

RECOMMENDATIONS TO HELP PROTECT YOUR FAMILY

BE+ AGAINST COVID

We know you worry each time you go to work because you think you will transmit the coronavirus to your family. Here, we provide some recommendations to help reduce the risk of a possible infection by the SARS-CoV-2 (COVID-19).

Don't wait to have symptoms to put them into practice

ESTABLISH SOME PATTERNS:

- Take the minimum number of objects to work: keys, mobile phone, wallet, glasses, handbag, backpack.
- Whenever possible take a shower before leaving your work place.
- Try that all material used at work remain in the centre for cleansing and disinfection (clothes and shoes).

WHEN YOU ARRIVE HOME:

- Don't touch anything.
- Take off your shoes and leave them at the door.
- Clean the objects you will use while at home with alcohol at a 70% concentration: mobile phone, glasses…
- Place the clothes brought from work in a separate closed bag until you put the to wash.
- Take a shower immediately.

GENERIC RECOMMENDATIONS:

- Wash your hands frequently with water and soap or alcohol-based solutions.
- Cover your mouth and nose with your bent elbow when coughing or sneezing.
- Use disposable tissues.
- Avoid touching your eyes, nose and mouth.
- Try not to share the bed or the room with other people.
- Be very vigilant of the appearance of symptoms.

IF YOU ARE LIVING WITH PEOPLE AT RISK:

- Establish minimum contact with that person.
- Use facemask if you have to help them and wash your hands as many times as possible.
- Use separate bathrooms and/or disinfect after each use.

HOME CLEANING:

- Wash personal clothes, bed linen and towels in the washer at 60 – 90 ºC (140 – 194 ºF) using your regular detergent. There is no need to separate the laundry. Do not shake the clothes.
- Use disposable gloves and other material.
- Clean the kitchen and bathrooms with a 1:50 solution of household bleach.
- Clean high-touch surfaces with a 1:50 solution of household bleach: knobs, handles, taps, switches, phones, computers, keyboards…
- Preferably use waste bins with a pedal and a lid to dispose of material used for cleaning.
- Do not share personal items: toothbrush, drinking glasses, utensils, towels…
- Wash dishes and utensils in a dishwasher or with hot water and soap
- Keep common spaces ventilated.

COVID-19

Junta de Andalucía
Consejería de Salud y Familias
SERVICIO ANDALUZ DE SALUD

HOSPITAL UNIVERSITARIO CLÍNICO SAN CECILIO

HOW TO BEGING THE DAILY ACTIVITIES IN AN INTENSIVE CARE UNIT SHIFT

At the beginning of the shift, each professional must enter the unit with:

The regulation uniform
Enclosed footwear
Surgical facemask

Once in the care area, the first thing the professional will undergo is a BRIEFING

Duration: 10-15 minutes
Aimed to all personnel in every room: intensive care medical specialists, resident medical interns, nurses, nursing care assistant technicians and safety coordinators

INFORMATION TO BE DELIVERED IN THE BREFING:

1. Availability and indications for the different types of equipment*:

FACEMASK

Surgical facemask → All the time and in any area of the ICU except when the following is indicates:

Facemask FFP2 for distances under two meters with the patients.

Facemask FFP3 for procedures with high-risk of generating aerosol at high concentrations. Basically reserved for orotracheal intubation.

PERSONAL PROTECTIVE EQUIPMENT

For any procedure in de the room

- Fluid-resistant gown
- Shoe covers
- Cap
- Goggles and/or visor
- Facemask FFP2
- Double layer of gloves

For an orotracheal intubation and pronation-depronation

- Coverall
- Shoe covers
- Goggles and/or visor
- Facemask FFP2
- Facemask FFP3 (only for the person performing orotracheal intubation)
- Double layer of gloves

GLOVES

Indicated:

- As part of the standard precautions during a procedure when there is perceived risk of exposure to bodily fluids.
- Gloves must also be used when assisting colleagues that are using the above-mentioned personal protective equipment

Remember:

- Perform hand hygiene before putting on the gloves
- Remove gloves immediately after finishing the task
- Perform hand hygiene after removing gloves

In all other situations, the use of gloves is counterproductive. They macerate and spoil the hands and eliminate the perception of risk. Hands with gloves get contaminated as much or more than hands without gloves.

2. Specific briefing on the situation of each patient. ISOBAR / SBAR

3. Allocation of patients and day planning

Include a positive news

*Following the recommendation of the document titled "*PROCEDIMIENTO DE ACTUACIÓN PARA LOS SERVICIOS DE PREVENCIÓN DE RIESGOS LABORALES FRENTE A LA EXPOSICIÓN AL NUEVO CORONAVIRUS* (SARS-CoV-2)" (ACTION PROTOCOL FOR THE PREVENTION OF OCCUPATIONAL HAZARDS SERVICES AGAINST THE EXPOSURE TO THE NOVEL CORONAVIRUS (SARS-CoV-2)) prepared by the Ministry of Health on March 24, 2020

COVID-19

Junta de Andalucía
Consejería de Salud y Familias
SERVICIO ANDALUZ DE SALUD

HOSPITAL UNIVERSITARIO CLÍNICO SAN CECILIO

Care for professionals with acute stress disorder

Intensive Care Unit

BRIEFING

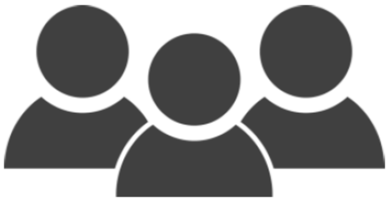

PURPOSE

Strengthen the safety and help control the pressure professionals are under, with specific focus on new hires.

WHERE	WHEN	DURATION
In each ROOM	At the beginning of each shift	10-15 minutes

WHO

- Intensive care medical specialists
- Resident medical interns
- Nurses
- Nursing care assistant technicians
- Safety coordinator

CONTENT

Regarding professionals:

✓ All have the necessary equipment

✓ Key aspects for using personal protective equipment (PPE)

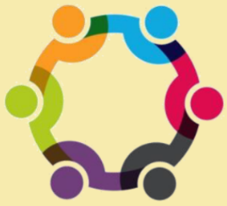

Regarding patients:

✓ SBAR/ISOBAR

✓ Key points

✓ Allocation of patients and day planning

Always include at least one good news

None of Us is as Good as All of Us *Ray Kroc*

https://secondvictimscovid19.umh.es/

COVID-19

Junta de Andalucía
Consejería de Salud y Familias
SERVICIO ANDALUZ DE SALUD

HOSPITAL UNIVERSITARIO CLÍNICO SAN CECILIO

Care for professional with acute stress

Intensive Care Unit

RECOVERY PAUSES

PURPOSE

Offer regulated recovery periods during the workday using recovery and self-control tools.

WHEN – Twice every shift

Room	Morning		Afternoon		Night	
**A and B**	10h30	12h30	18h	20h15	02h	05h
**C**	10h45	12h45	18h15	20h30	02h15	05h15

FOR WHOM

All the staff in a room will take the rest together, with two people remaining on duty in the other room.

- ✓ **Two professionals from room C in pauses of rooms A and B**
- ✓ **Two professionals from rooms A and B in pauses of room C**

DURATION

5-7 minutes

HOW?

In the meditation room

- ✓ **Closed doors**
- ✓ **Level of light attenuated**
- ✓ **Silence**

Listen to the audio. It will help you relax

None of Us is as Good as All of US. Ray Kroc

BE+ AGAINST COVID

https://secondvictimscovid19.umh.es/p/home.html

COVID-19

Junta de Andalucía
Consejería de Salud y Familias
SERVICIO ANDALUZ DE SALUD

HOSPITAL UNIVERSITARIO CLÍNICO SAN CECILIO

Support service for taking care of emotional health of our professionals

As healthcare workers, we are used to deal with alert situations on a daily basis and have acquired multiple personal resources, in addition to strengthening our ability to recover (resilience). However, we are humans and may undergo emotional issues during the pandemic crisis caused by the coronavirus (COVID-19).

The San Cecilio Clinical Hospital (Granada, Spain) offers this additional support for the **care of the emotional health of our professionals** and help them cope with emotional situations.

Key principles to regulate our emotions

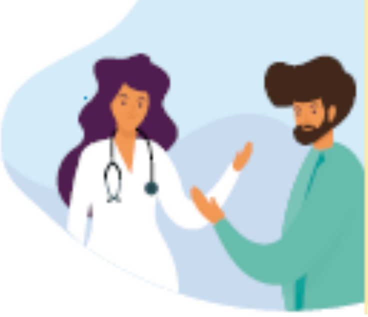

1
Share your emotions with family and friends. Talk to them, they will listen and support you.

2
Your co-workers will understand you better than anyone else. Communicate with them. Peer support is a powerful tool, use it!

Relaxation tools

Sometimes, basic measures are not enough and alert situations are not always relieved this way. A large number of meditation and relaxation audios are available in the Internet, but when we arrive home from a day of work we are too tired to decide which to choose.

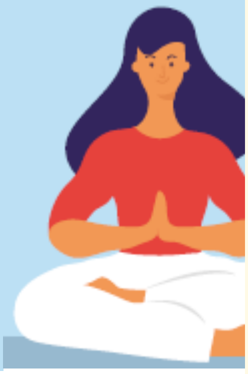

Basic tips for an emotional balance

1 Sleep

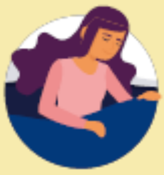

Good eating habits 2

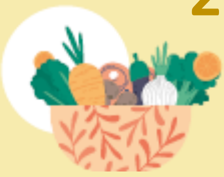

3 Rest

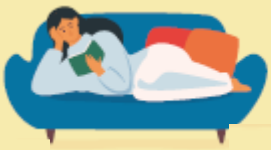

4

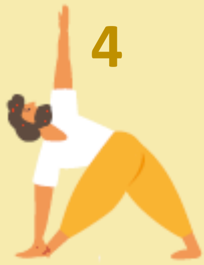

Moderate-intensity exercise

5

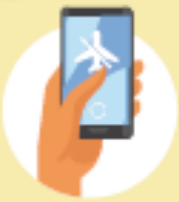

Disconnect from time to time (avoid being over-informed)

Contact channels for personalized care

Corporate email:
cuidaralprofesional.hucsc.sspa@juntadeandalucia.es

Information you must include in your email:
1. Name
2. Contact telephone number or email address
3. In few words tell us what you need and describe your symptoms

We will contact you
Telephone: **693704** (corporate) or **671593704**
Generally from Monday to Friday between 9:00 a.m. and 2:00 p.m.

If you experience symptoms such as intense anxiety, insomnia, or disproportionate rage and none of the above-recommended basic tools help you

Contact Us!

RED ANDALUZA DE servicios sanitarios y espacios LIBRES DE HUMO

@clinicodegranada www.clinicodegranada.es
