# Supplementary material for: Preventing and Addressing the Stress Reactions of Health Care Workers Caring for Patients With COVID-19: Development of a Digital Platform (Be + Against COVID)
Source: JMIR Mhealth Uhealth. 2020 Oct 5;8(10):e21692. doi: 10.2196/21692 (PMC7537725; doi:10.2196/21692)
Supplement: Multimedia Appendix 3 [file mhealth_v8i10e21692_app3.docx]

# Development of the platform “Be+ against COVID”, a website and a mobile app to prevent and address the stress reactions of healthcare workers caring COVID-19 patients

Jose Joaquín Mira^1,2,4,5^, María Asunción Vicente^3,5^, Adriana López-Pineda^1,4^, Irene Carrillo^1,4,5^, Mercedes Guilabert^1,5^, César Fernández^3,5^, María Virtudes Pérez-Jover^1,5^, Jimmy Martín-Delgado^4^, Pastora Pérez-Pérez^6^, Ángel Cobos-Vargas^7^, María Pilar Astier-Peña^8^, Olga Beatriz Martínez-García^7^, Bárbara Marco^9^, Cristina Abad^9^ on behalf of SARS-CoV-2 Second Victim Study Group*

From the ^1^Department of Health Psychology, Miguel Hernández University, Elche, Spain; ^2^Salud Alicante-Sant Joan Health District, Alicante, Spain; ^3^Department of Systems Engineering and Automation, Miguel Hernández University, Elche, Spain;

^4^FISABIO, Alicante, Spain; ^5^Prometeo/2017/173 Excellence Group from Generalitat Valenciana; ^6^Patient Safety Observatory, Seville, Spain; ^7^Hospital Universitario Clínico San Cecilio de Granada, Spain; ^8^Centro de Salud “La Jota”, Zaragoza, Spain; ^9^Zona de salud de Calatayud, Zaragoza, Spain;

*Collaborators

SARS-CoV-2 Second Victim Study Group is comprised of Mª José Bueno Domínguez, (Grup SAGESSA, Reus), Julián Vitaller Burillo, Juan Francisco Herrera Cuenca, (Inspección Médica, Alicante), Antonio Guilabert Giménez (Hospital de Yecla, Servicio Murciano de Salud, Yecla), Matilde Lahera Martín, Carmen Silvestre Bustos (Osasunbidea, Pamplona), Susana Lorenzo Martínez (Hospital Universitario Fundación Alcorcón, Madrid), Ascensión Sánchez Martínez (Hospital General Universitario Reina Sofía, Murcia), María Luisa Torijano Casalengua (Servicio de Salud Castilla-La Mancha, Toledo), Carolina Varela Rodríguez (Hospital 12 de Octubre), Auxiliadora Javaloyes, Inmaculada Palazón, José Navarro (Hospital General Universitario de Alicante), Carlos Aibar Remón (Hospital Clínico Universitario Lozano Blesa, Zaragoza) and Jesús M. Aranaz Andrés (Hospital Universitario Ramón y Cajal, Madrid).
